# Parkinson disease treatments on national essential medicines lists, African Region

**DOI:** 10.2471/BLT.25.293460

**Published:** 2025-08-25

**Authors:** Natasha Fothergill-Misbah, Rodrigo Cataldi, Mark Stothard, Neerja Chowdhary, Njideka Okubadejo, Bernadette Cappello, Richard Walker, Tarun Dua

**Affiliations:** a Population Health Sciences Institute, Newcastle University, Newcastle upon Tyne, England.; b Department of Mental Health, Brain Health and Substance Use, World Health Organization, 20 Avenue Appia, 1211 Geneva 27, Switzerland.; c Northumbria Healthcare NHS Foundation Trust, North Shields, England.; d College of Medicine, University of Lagos, Lagos, Nigeria.; e Department of Health Products Policy and Standards, World Health Organization, Geneva, Switzerland.

## Abstract

The prevalence of Parkinson disease is increasing globally. Despite the existence of effective and affordable medicines for Parkinson disease, access to these medicines is limited in the World Health Organization (WHO) African Region. Several factors influence accessibility, including lack of prioritization of Parkinson disease, shortage of a trained health workforce, barriers to health financing and lack of inclusion of medicines in national essential medicines lists. We determined alignment of the most recent national essential medicines lists of 47 countries in the WHO African Region with the 23rd edition of the *WHO Model list of essential medicines* for Parkinson disease medicines. Overall, of any formulation or strength, 81% (38/47) of countries included levodopa + carbidopa or levodopa + benserazide as a therapeutic alternative on their national lists; and 79% (37/47) included biperiden or trihexyphenidyl as a therapeutic alternative. Inclusion of specific formulations for medicines was lower; for example, 45% (21/47) of countries included levodopa + carbidopa or levodopa + benserazide in a 4:1 ratio. Furthermore, 11% (5/47) of national essential medicines lists included none of the four medicines. While inclusion of medicines for Parkinson disease in national essential medicines lists provides no guarantee of immediate access, it can encourage procurement, prescribing and use, and can help lower costs, raise awareness of and create political will for Parkinson disease treatment. This analysis provides further evidence of the need for action to improve the accessibility of medicines for Parkinson disease in the WHO African Region.

## Résumé

La prévalence de la maladie de Parkinson augmente dans le monde entier. Malgré l’existence de médicaments efficaces et abordables pour la traiter, l’accès à ces médicaments est limité dans la région de l’Afrique de l’Organisation mondiale de la Santé (OMS). Plusieurs facteurs influencent cette accessibilité, notamment le manque de priorité accordée à la maladie de Parkinson, la pénurie de personnel de santé qualifié, les obstacles au financement de la santé et l’absence d’inclusion des médicaments dans les listes nationales de médicaments essentiels. Nous avons déterminé la conformité des listes nationales de médicaments essentiels les plus récentes de 47 pays de la région d’Afrique de l’OMS, avec la 23^e^ édition de la *Liste modèle de l’OMS des médicaments essentiels* pour la maladie de Parkinson. Dans l’ensemble, toutes formulations et toutes concentrations confondues, 81% (38/47) des pays ont inclus la lévodopa + carbidopa ou la lévodopa + bensérazide comme alternative thérapeutique dans leurs listes nationales et 79% (37/47) ont inclus le biperidène ou le trihexyphénidyle comme alternative thérapeutique. L’inclusion de formulations spécifiques pour les médicaments était moins fréquente. Par exemple, 45% (21/47) des pays ont inclus la lévodopa + carbidopa ou la lévodopa + bensérazide dans un rapport de 4:1. En outre, 11% (5/47) des listes nationales de médicaments essentiels ne reprenaient aucun des quatre médicaments. Si l’inclusion de médicaments pour la maladie de Parkinson dans les listes nationales de médicaments essentiels ne garantit pas un accès immédiat, elle peut encourager l’achat, la prescription et l’utilisation de ces médicaments et contribuer à réduire les coûts, à sensibiliser le public et à créer une volonté politique en faveur du traitement de la maladie de Parkinson. La présente analyse apporte une preuve supplémentaire de la nécessité d’agir pour améliorer l’accessibilité des médicaments pour la maladie de Parkinson dans la région d’Afrique de l’OMS.

## Resumen

La prevalencia de la enfermedad de Parkinson está aumentando a nivel mundial. A pesar de la existencia de medicamentos eficaces y asequibles para esta enfermedad, el acceso a los mismos es limitado en la Región de África de la Organización Mundial de la Salud (OMS). Diversos factores influyen en esta falta de acceso, entre ellos, la escasa priorización de la enfermedad de Parkinson, la escasez de personal sanitario capacitado, las barreras en la financiación sanitaria y la ausencia de inclusión de los medicamentos en las listas nacionales de medicamentos esenciales. Se determinó el grado de alineación de las listas nacionales de medicamentos esenciales más recientes de 47 países de la Región de África de la OMS con la 23.ª edición de la *Lista modelo OMS de medicamentos esenciales*, en lo que respecta a los medicamentos para la enfermedad de Parkinson. En términos generales, independientemente de la formulación o concentración, el 81% (38/47) de los países incluían levodopa + carbidopa o levodopa + benserazida como alternativas terapéuticas en sus listas nacionales y el 79% (37/47) incluían biperideno o triexifenidilo. La inclusión de formulaciones específicas fue menor; por ejemplo, solo el 45% (21/47) de los países incluían levodopa + carbidopa o levodopa + benserazida en una proporción 4:1. Además, el 11% (5/47) de las listas nacionales de medicamentos esenciales no incluían ninguno de los cuatro medicamentos analizados. Aunque la inclusión de medicamentos para la enfermedad de Parkinson en las listas nacionales no garantiza su acceso inmediato, puede facilitar su adquisición, prescripción y uso, además de contribuir a la reducción de costos, aumentar la concienciación y generar voluntad política para el tratamiento de esta enfermedad. Este análisis aporta nuevas pruebas de la necesidad urgente de mejorar el acceso a medicamentos para la enfermedad de Parkinson en la Región de África de la OMS.

## ملخص

يزداد انتشار مرض باركنسون على الصعيد العالمي، وعلى الرغم من وجود أدوية فعالة وبأسعار في المتناول لعلاجه، إلا أن إمكانية الحصول على هذه الأدوية محدود في المنطقة الأفريقية التابعة لمنظمة الصحة العالمية (WHO). وتؤثر عدة عوامل على إمكانية الحصول هذه، ومنها عدم منح الأولوية لمرض باركنسون، والنقص في القوى العاملة الصحية المدربة، وعوائق تمويل الرعاية الصحية، وعدم إدراج الأدوية في قوائم الأدوية الأساسية الوطنية. لقد قمنا بتحديد مدى توافق أحدث قوائم الأدوية الأساسية الوطنية لعدد 47 دولة في المنطقة الأفريقية التابعة لمنظمة الصحة العالمية مع الإصدار 23 من القائمة النموذجية لمنظمة الصحة العالمية للأدوية الأساسية لأدوية مرض باركنسون. وبشكل عام، أدرجت %81 (38/47) من الدول، من أي تركيبة أو تركيز، ليفودوبا + كاربيدوبا، أو ليفودوبا + بنسيرازيد، كبديل علاجي في قوائمها الوطنية، بينما أدرجت %79 (37/47) بيبيريدين، أو تريهيكسفينيديل كبديل علاجي. كان إدراج تركيبات محددة للأدوية أقل؛ على سبيل المثال، أدرجت %45 (21/47) من الدول ليفودوبا + كاربيدوبا، أو ليفودوبا + بنسيرازيد، بنسبة 4:1. علاوة على ذلك، لم تتضمن %11 (5/47) من قوائم الأدوية الأساسية الوطنية أيًا من الأدوية الأربعة. وبالرغم من أن إدراج أدوية مرض باركنسون في قوائم الأدوية الأساسية الوطنية لا يقدم أية ضمانات بالحصول عليها بشكل فوري، إلا أنه قد يشجع على الشراء والوصف والاستخدام، ويمكن أن يساعد في خفض التكاليف، وزيادة الوعي بعلاج مرض باركنسون، وتهيئة إرادة سياسية لهذا العلاج. يقدم هذا التحليل دليلًا إضافيًا على الحاجة لاتخاذ إجراء لتحسين إمكانية الحصول على أدوية مرض باركنسون في المنطقة الأفريقية التابعة لمنظمة الصحة العالمية.

## 摘要

帕金森病的全球患病率正在逐步上升。尽管市面上已有适用于治疗帕金森病的各种有效且价格实惠的药物，但是在世卫组织 (WHO) 非洲区域，此类药物的获取渠道十分有限。影响可获性的因素有很多，包括对帕金森病不够重视、受过培训的卫生人力短缺、卫生筹资障碍以及对症药物未被纳入《国家基本药物清单》中。我们确定，世卫组织非洲区域 47 个国家的最新版《国家基本药物清单》中列出的帕金森病治疗药物与第 23 版世卫组织《基本药物标准清单》中列出的此类药物保持一致。总体而言，在不考虑剂型或规格的前提下，有 81% (38/47) 的国家将左旋多巴+卡比多巴或者左旋多巴+苄丝肼作为替代治疗药物纳入了其《国家基本药物清单》中，且有 79% (37/47) 的国家将比哌立登或者苯海索作为替代治疗药物纳入了此类清单中。特定剂型药物的纳入比例较低，例如：45% (21/47) 的国家按照 4:1 的比例纳入了左旋多巴+卡比多巴或者左旋多巴+苄丝肼。此外，11% (5/47) 的国家的《国家基本药物清单》中未纳入以上四种药物中的任何一种。尽管将帕金森病治疗药物纳入《国家基本药物清单》中并不能保证患者可立即获得此类药物，但这一举措却能推动此类药物的采购、处方开具和使用，并有助于降低帕金森病治疗费用、提高治疗意识以及促进相关政治意愿的形成。本次分析进一步证明了需要采取行动以提高世卫组织非洲区域内帕金森病治疗药物的可获性。

## Резюме

Распространенность болезни Паркинсона растет во всем мире. Несмотря на наличие эффективных и доступных средств лечения болезни Паркинсона, доступ к этим лекарственным препаратам в Африканском регионе Всемирной организации здравоохранения (ВОЗ) ограничен. На доступность влияют несколько факторов, в том числе недостаточная приоритетность болезни Паркинсона, нехватка обученного медперсонала, препятствия на пути финансирования здравоохранения и недостаточная включенность таких препаратов в национальные перечни основных лекарственных средств. Авторы определили, в какой мере национальные перечни основных лекарственных средств 47 стран Африканского региона ВОЗ соответствуют 23-му изданию *Примерного перечня основных лекарственных средств ВОЗ* в отношении препаратов для лечения болезни Паркинсона. В целом из всех рецептур и концентраций в качестве терапевтической альтернативы национальные перечни включали сочетание леводопы с карбидопой или леводопы с бенсеразидом в 81% (38/47) стран и бипериден или триэксилфенидил в 79% (37/47) стран. Включение определенных рецептур лекарственных средств было более низким. Так, например, в 45% (21/47) стран в перечни входили сочетания карбидопы с леводопой или леводопы с бенсеразидом в соотношении 4:1. Кроме того, в 11% случаев (5/47) в национальные перечни основных лекарственных средств не входил ни один из этих четырех препаратов. Несмотря на то что включение препаратов для лечения болезни Паркинсона в национальные перечни основных лекарственных средств не гарантирует непосредственного доступа к ним, это может способствовать их закупкам, назначению и использованию, а также помочь снизить затраты, повысить уровень осведомленности и создать политическую волю для лечения болезни Паркинсона. Анализ предоставляет дополнительные свидетельства потребности в действиях с целью улучшить доступность лекарств для лечения болезни Паркинсона в Африканском регионе ВОЗ.

## Introduction

Parkinson disease is a progressive neurodegenerative condition with an estimated worldwide prevalence of 12 million people. The disease is a leading cause of disability among neurological disorders.^1^ The true burden of Parkinson disease, however, is difficult to estimate due to low rates of diagnosis and limited epidemiological evidence across the world.^2^^–^^4^ The main risk factor for Parkinson disease is increasing age, although incidence is also affected by genetic predispositions and exposure to pesticides.^5^ These factors, particularly the environmental factors, are increasingly putting both urban and rural populations in low- and middle-income countries, such as those countries in the World Health Organization (WHO) African Region, at greater risk of developing Parkinson disease.^6^^,^^7^

Despite the ageing global population and evidence of increasing Parkinson disease prevalence,^8^ health systems in low- and middle-income countries are not prepared to diagnose and manage the disease. This situation is in part due to a lack of specialist neurological services in many low- and middle-income countries, leading to underdiagnosis and an underestimation of the disease burden.^9^^,^^10^ Lack of awareness and stigma associated with Parkinson disease^11^^–^^14^ can further delay diagnosis and therefore access to effective and appropriate treatment. Combined, these factors limit policy prioritization in low- and middle-income countries and the burden of disease remains unrecognized. As a result, large proportions of the population in these countries are without access to treatment.

Medication for Parkinson disease can be life changing. Levodopa + dopa decarboxylase inhibitor (that is, carbidopa or benserazide) is the most effective medicine for improving symptoms, functioning and quality of life.^15^^,^^16^ Anticholinergics (that is, biperiden or trihexyphenidyl), which act via a different therapeutic mechanism, are also beneficial in Parkinson disease treatment, particularly for tremor. Despite their proven efficacy, poor accessibility of these medicines in low- and middle-income countries is common and a key contributor to the treatment gap. Where medicines are available, they are often unaffordable,^10^ especially in the WHO African Region. For example, levodopa + carbidopa is unaffordable in Ghana,^17^ Kenya^18^ and Nigeria,^19^ with availability in pharmacies ranging from 11% (13/121) in Ghana to 50% (24/48) in Kenya. According to WHO, only 34% (37/110) of countries report availability of levodopa + carbidopa at all times at the primary care level.^10^

Many different factors affect access to medicines, for example: shortage of a trained health workforce able to diagnose and prescribe medicines; lack of access to universal health coverage (UHC); and inefficiency of supply chains.^20^ A key component that negatively affects access to medicines for Parkinson disease is the poor prioritization or selection of essential medicines,^20^ which are not included on national essential medicines lists.

The *WHO Model list of essential medicines*^21^ includes a selection of medicines that satisfy the priority health-care needs of a population. Essential medicines should be available within the context of functioning health systems at all times, in adequate amounts and appropriate dosage forms, with assured quality, and at an affordable price.^22^ The WHO model list is updated every two years, and acts as a guide for countries or regional authorities to develop or revise their own national essential medicines lists based on local health priorities and treatment guidelines. Selecting a limited number of essential medicines that reflect the national disease burden and clinical need can improve access by streamlining procurement and distribution of quality-assured medicines. This selection also encourages more rational prescribing, enhances appropriate use, and helps reduce costs for health-care systems and patients.^23^ National essential medicines lists have also been successful in raising awareness and political will in resource-constrained countries.^23^ However, due to the many different factors influencing access to medicines, inclusion on national essential medicines lists does not automatically mean these medicines will be available or affordable.^20^

The disease burden due to neurological disorders such as Parkinson disease cannot be reduced without reliable and equitable access to essential medicines. The imperative to increase access to essential medicines was highlighted in the United Nations sustainable development goals (goals 3.4 and 3.8) and in the *Intersectoral global action plan on epilepsy and other neurological disorders 2022–2031*.^24^

As the accessibility of medicines is determined by several factors, different methods have been used to evaluate access, for example, direct availability and pricing through facility-based surveys,^18^^,^^19^ stakeholder interviews^25^^–^^27^ or direct government reporting.^10^^,^^28^ Analysing national essential medicines lists offers insights into key barriers to access. Therefore, the aim of this analysis was to determine the alignment of Parkinson disease medicines listed in national essential medicines lists of countries in the WHO African Region with those medicines in the 23rd list of the *WHO Model list of essential medicines*.^21^

## Methods

**Fig. 1:**
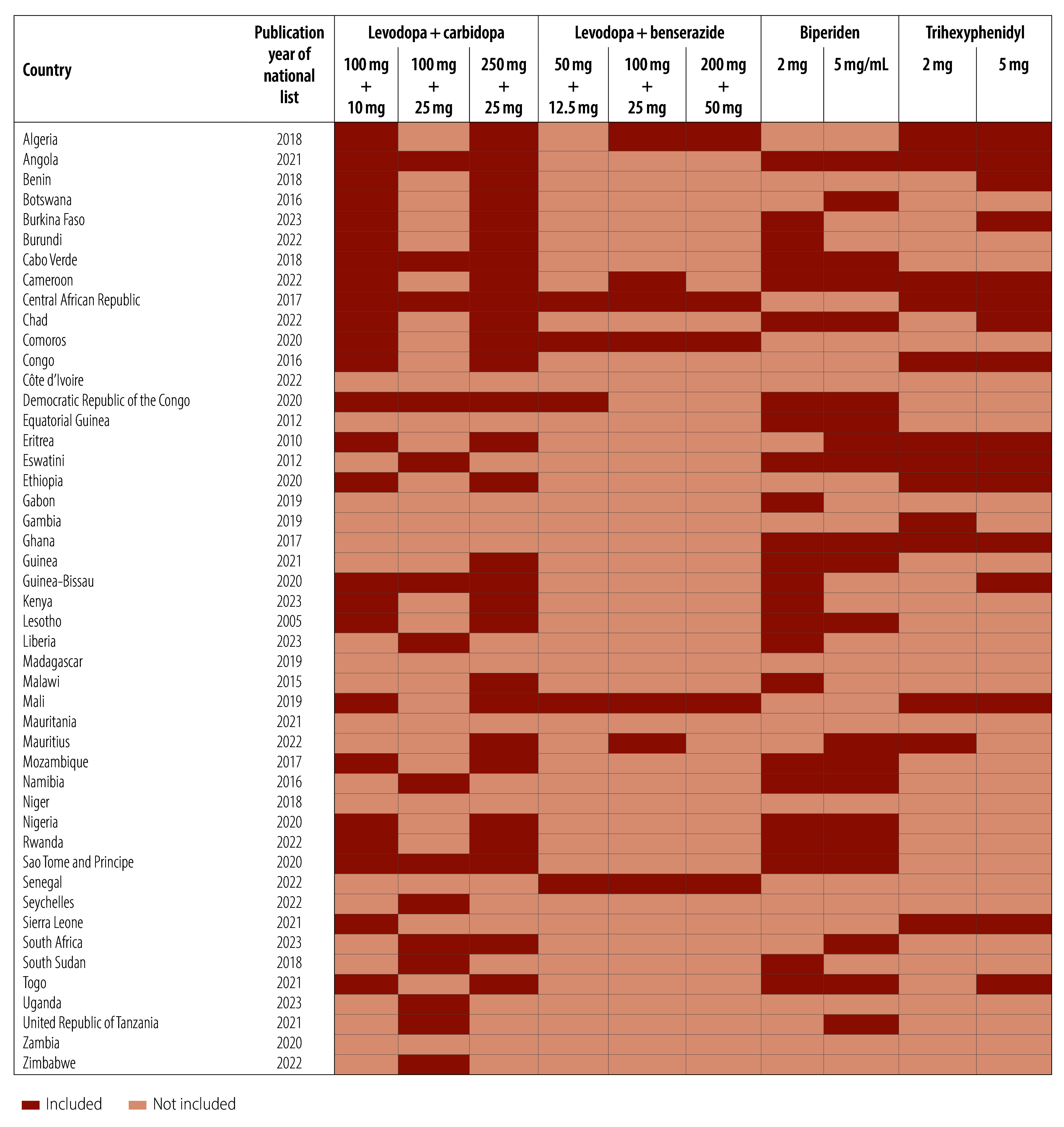
Medicines for Parkinson disease on the WHO essential medicines list that are included in national lists, by country, WHO African Region

We obtained digital versions of national essential medicines lists for 47 countries (Fig. 1) in June 2024 through: (i) web-based internet searches of Google (search terms included country name + essential medicine* list (in English, French, Portuguese and Spanish)); or (ii) directly through local representatives via WHO. We downloaded the national lists and securely stored them in a shared folder for analysis. All the latest versions of national essential medicines lists for the WHO African Region (including PDF files) are now available on a WHO repository.^29^ On re-searching the repository in May 2025, we found updated national essential medicines lists for six countries.

WHO: World Health Organization.

To ensure accuracy of data extraction, two people independently recorded in Excel (Microsoft, Redmond, United States of America) all medicines for Parkinson disease listed on the WHO essential medicines list and national essential medicines lists, including specific formulations and strengths. For all national list documents, including in other languages (i.e. French, Portuguese and Spanish), we used the term “parkinson” to identify the required sections (e.g. “medicines for parkinsonism,” “antiparkinsonicos,” “antiparkinsoniens”). We also made manual searches. We determined the proportion of essential medicines and individual formulations included on national essential medicines lists compared with the WHO essential medicines list. We used the square box listings in the WHO essential medicines list, which indicates that medicines within a pharmacological class can be considered therapeutically equivalent in terms of efficacy and safety.^30^ Selecting a single medicine from within a pharmacological class of therapeutically equivalent medicines can result in better-value procurement, improved access and more rational prescribing.^23^

The medicines for Parkinson disease listed on the 2023 WHO essential medicines list are: (i) biperiden (2 mg; 5 mg/1 mL) or trihexyphenidyl as a therapeutic alternative; and (ii) levodopa + carbidopa (100 mg + 10 mg; 100 mg + 25 mg; 250 mg + 25 mg) or levodopa + benserazide as a therapeutic alternative.

We also report medicines for Parkinson disease that are included on national essential medicines lists, but not on the WHO list.

## Results

We analysed data from the national essential medicines lists of 47 countries in WHO’s African Region. Fig. 1 shows the year of publication of the latest national list for each country and which medicines for Parkinson disease were included. Of note, the WHO essential medicines list does not include specific formulations for therapeutic alternatives (i.e. levodopa + benserazide and trihexyphenidyl). However, all national essential medicines lists did include specific formulations and we therefore report on these formulations.

Of the 47 countries, four (9%) had not updated their national essential medicines lists since before 2015, 16 (34%) most recently updated their lists between 2015 and 2019, while 27 (57%) countries last updated their lists between 2020 and 2023.

Only the national essential medicines lists of Cameroon and Mauritius included all four medicines in WHO’s essential medicines list. In contrast, the national lists of Côte d’Ivoire, Madagascar, Mauritania, Niger and Zambia did not include any of the four Parkinson disease medicines. Given the square box concept, 81% (38/47) of countries included levodopa + carbidopa or levodopa + benserazide as a therapeutic alternative (of any formulation or strength) on their national lists (Table 1). Similarly, 79% (37/47) of countries included biperiden or trihexyphenidyl as a therapeutic alternative (of any formulation or strength) on their national lists.

**Table 1:** Medicines for Parkinson disease included on countries’ national essential medicines lists, WHO African Region

Medicine (any strength) included^a^	No. of countries (%) (*n* = 47)
**Levodopa + carbidopa or levodopa + benserazide**	
Yes	38 (81)
No	9 (19)
**Biperiden or trihexyphenidyl**	
Yes	37 (79)
No	10 (21)

^a^ Based on square box listing.^30^

As individual medicines, levodopa + carbidopa (any formulation or strength) was included in 79% (37/47) of national essential medicines lists. Levodopa + benserazide had the lowest overall inclusion at 17% (8/47), followed by trihexyphenidyl at 38% (18/47) and biperiden at 62% (29/47; Table 2 and Table 3). In terms of strength, only 32% (15/47) of countries included levodopa + carbidopa 100 mg + 25 mg on their national essential medicine lists (Table 2). Levodopa + benserazide 100 mg + 25 mg was the most common strength of this medicine included on  the national lists of countries (15%; 7/47), although only 17% (8/47) of national lists included this medicine at all (Table 2). Biperiden 2 mg tablets and 5 mg/mL injection were included on 51% (24/47) and 45% (21/47) of national essential medicines lists respectively (Table 3). Trihexyphenidyl 2 mg tablets were included in 28% (13/47) of national essential medicines lists and 5 mg tablets were included in 34% (16/47; Table 3).

**Table 2:** Strengths of levodopa combinations included on countries’ national essential medicines lists, WHO African Region

Medicine	No. of countries (%) (*n = 47)
**Levodopa + carbidopa**	
Any strength	37 (79)
100 mg + 10 mg	25 (53)
100 mg + 25 mg	15 (32)
250 mg + 25 mg	28 (60)
**Levodopa + benserazide** ^a^	
Any strength	8 (17)
50 mg + 12.5 mg	5 (11)
100 mg + 25 mg	7 (15)
200 mg + 50 mg	5 (11)

^a^ Therapeutic alternative.^30^

**Table 3:** Strengths of biperiden and trihexyphenidyl included on countries’ national essential medicines lists, WHO African Region

Medicine	No. of countries (%) (*n* = 47)
**Biperiden**	
Any strength	29 (62)
2 mg	24 (51)
5 mg/mL	21 (45)
**Trihexyphenidyl^a^**	
Any strength	18 (38)
2 mg	13 (28)
5 mg	16 (34)

^a^ Therapeutic alternative.^30^

Twenty-three medicines for Parkinson disease (individual medicines, including tablets and injections and varying strengths) were listed on the countries’ national essential medicines lists that were not listed on the WHO list. The reasons why these medicines were not included on the WHO list are shown in Table 4. Overall, 66% (31/47) of countries had at least one medicine on their national essential medicines list that is not included on the WHO list. The most notable was bromocriptine, which was included on 19% (9/47) of national essential medicines lists, followed by tropatepine on 11% (5/47) and benztropine on 9% (4/47).

**Table 4:** Medicines listed on countries’ national essential medicines lists for Parkinson disease not listed on the WHO list for this condition, WHO African Region

Medicine and formulation	Countries listing the medicine	Details
Benztropine 2 mg tablet	Ethiopia, Ghana	No application made to the WHO list for Parkinson disease
Benztropine mesylate 1 mg/mL injection	Gambia, South Sudan	No application made to the WHO list for Parkinson disease
Biperiden 4 mg tablet	Burkina Faso, Guinea, Guinea-Bissau, Sao Tome and Principe	Medicine included in the WHO list in alternative strength or formulation
Biperiden 0.4 mg/mL oral solution	Angola	Medicine included in the WHO list in alternative strength or formulation
Bromocriptine 2 mg tablet	Côte d’Ivoire	Medicine included in the WHO list in alternative strength or formulation for another indication Application for inclusion on the WHO list for Parkinson disease rejected in 2015
Bromocriptine 2.5 mg tablet	Botswana, Burkina Faso, Eswatini, Namibia, United Republic of Tanzania, Zambia	Medicine included in the WHO list for another indication Application for inclusion on the WHO list for Parkinson disease rejected in 2015
Bromocriptine 5 mg tablet	Côte d’Ivoire, Sao Tome and Principe	Medicine included in the WHO list for another indication Application for inclusion on the WHO list for Parkinson disease rejected in 2015
Bromocriptine 10 mg tablet	Algeria	Medicine included in the WHO list in alternative strength or formulation for another indication Application for inclusion on the WHO list for Parkinson disease rejected in 2015
Levodopa + benserazide 250 mg + 25 mg tablet	Mauritania	This strength does not exist
Orphenadrine 50 mg tablet	Malawi, Mauritius, Zimbabwe	No application made to the WHO list for Parkinson disease or any other indication
Orphenadrine 100 mg tablet	Mauritius	No application made to the WHO list for Parkinson disease or any other indication
Pramipexole 180 µg tablet	Kenya	Application for inclusion of alternative strength or formulation on the WHO list for Parkinson disease rejected in 2015
Pramipexole 700 µg tablet	Kenya	Application for inclusion of alternative strength or formulation on the WHO list for Parkinson disease rejected in 2015
Piribedil 50 mg tablet	Benin, Côte d’Ivoire	No application made to the WHO list for Parkinson disease or any other indication
Procyclidine 5 mg tablet	Mauritius, Zambia	No application made to the WHO list for Parkinson disease or any other indication
Procyclidine 5 mg injection	Mauritius, Zambia	No application made to the WHO list for Parkinson disease or any other indication
Ropinirole 1mg tablet	Seychelles	Application for inclusion in the WHO list for Parkinson disease rejected in 2015
Selegiline 5 mg tablet	Congo, Sao Tome and Principe	No application made to the WHO list for Parkinson disease or any other indication
Trihexyphenidyl 10 mg injection	Benin, Liberia, Mali, Togo	Medicine included in the WHO list for Parkinson disease in alternative strength or formulation
Tropatepine 10 mg tablet	Benin, Comoros, Côte d’Ivoire	No application made to the WHO list for Parkinson disease or any other indication
Tropatepine 10 mg/2 mL injection	Guinea, Guinea-Bissau	No application made to the WHO list for Parkinson disease or any other indication
Valbenazine 40 mg tablet	Ethiopia	No application made to the WHO list for Parkinson disease or any other indication
Valbenazine 80 mg tablet	Ethiopia	No application made to the WHO list for Parkinson disease or any other indication

WHO: World Health Organization.

## Discussion

We explored the inclusion of medicines for Parkinson disease on national essential medicines lists in countries of the WHO African Region as absence from the list is a potential barrier to access. Levodopa + dopa decarboxylase inhibitor (carbidopa or benserazide) is still the mainstay of treatment worldwide, although several other classes of medicines are also available.^31^ The anticholinergics biperiden and trihexyphenidyl are rarely used in high-income countries due to their side-effects, but they are available in the WHO African Region at relatively low cost.^18^^,^^19^

Overall, when considering the WHO square box listing, alignment looks optimistic for any strength and formulation: 38 national essential medicines lists included levodopa + carbidopa or levodopa + benserazide, while 37 national essential medicines lists included biperiden or trihexyphenidyl. However, when considering individual strengths and formulations for specific medicines, alignment with the WHO essential medicines list is lower. For example, less than half of countries include levodopa + carbidopa or levodopa + benserazide in a 4:1 ratio (100 mg + 25 mg for carbidopa, or 50 mg + 12.5 mg, 200 + 50 mg for benserazide). While the WHO essential medicines list highlights the formulations, strengths and indications, it does not provide clinical guidance on how to use the medicines (i.e. prescribing practice, such as titration, dosing and dealing with side-effects). However, use of these formulations is recommended because they have a higher ratio of levodopa to dopa decarboxylase inhibitor, which results in fewer side-effects compared to a 10:1 ratio (i.e. 100 mg + 10 mg or 250 mg + 25 mg).^32^ The 4:1 formulation was also the least available in Kenya^18^ and Nigeria.^19^ Only two countries had all four recommended medicine groups included in their essential medicines lists, although this issue is not as concerning given the square box listing. Five countries had no medicines for Parkinson disease on their national essential medicine lists.

Although the inclusion of medicines for Parkinson disease in national essential medicines lists in the African Region is encouraging overall, gaps exist in alignment with the WHO list, which could be due to several reasons. First, some countries had not updated their national lists for several years, meaning they have not been reviewed in line with epidemiological shifts and the increasing burden of noncommunicable diseases and neurological disorders.^1^ The lower inclusion of some medicines (e.g. levodopa + benserazide) may also be explained by their more recent addition to the WHO essential medicines list (in 2021) and the lag in updating national lists. This situation could also result from a lack of resources in countries to conduct a comprehensive review of their national essential medicines lists across all diseases.^23^ Second, full awareness about Parkinson disease is lacking, even among policy-makers, including misconceptions that Parkinson disease is not common or that it is part of so-called normal ageing.^3^^,^^11^ Third, the shortage in a trained health workforce able to diagnose and manage Parkinson disease leads to a large diagnostic gap, making it seem as though Parkinson disease is rare. For example, in a community-based prevalence study in the United Republic of Tanzania, 78% (25/32) of people identified with Parkinson disease were previously undiagnosed.^33^ Fourth, limited data are available on the burden of Parkinson disease in the WHO African Region, which again makes it seem as though the disease is a rare disorder. These factors lead to a lack of prioritization of Parkinson disease and, consequently, suboptimal inclusion of medicines on national essential medicines lists.

Lack of inclusion of medicines on national essential medicines lists is a barrier to access. Essential medicines have been shown to be more available than other medicines in low- and middle-income countries globally, suggesting that inclusion on national essential medicines lists can influence availability.^34^ However, many different factors influence access and a comprehensive approach should be taken at the country level to improve accessibility.^20^ Furthermore, the inclusion of medicines on national essential medicines lists should be aligned with updated national standard treatment guidelines, and medicines should be registered with national regulatory authorities to increase efficiencies in procurement, safety and overall accessibility. 

To promote inclusion of Parkinson disease medicines on national essential medicines lists to increase accessibility of these medicines, other factors should be considered. First, the prevalence of Parkinson disease is expected to double in the next 30 years.^35^^,^^36^ However, awareness about Parkinson disease in the WHO African Region is low, which contributes to misconceptions that can result in stigma and discrimination.^11^ Furthermore, specialized neurological health workers are lacking and the few who are available are largely concentrated in cities.^10^^,^^25^ These factors are barriers to seeking medical care, leading to misdiagnosis or delayed diagnosis and therefore delayed treatment.

Second, Parkinson disease may not be viewed as a priority due to limited data on its burden and a perceived low return on investment. This situation may lead to little effort being made to update national essential medicines lists in accordance with the WHO list. However, the number of people living with Parkinson disease in Africa will increase,^8^ and updating national essential medicines lists could help with advocating for prioritization.

Third, the WHO essential medicines list serves as a guide for countries to develop and update their own national lists.^23^^,^^37^ However, selection should also be based on local epidemiological trends and priorities, which can be challenging when country-level data on burden of disease are lacking. A case study in the United Republic of Tanzania highlighted the different stakeholders and factors that are considered during the selection of medicines for the national essential medicines list in line with WHO’s recommendations, including efficacy and safety, availability, affordability and cost–effectiveness.^38^ The authors noted the lack of expertise in the evidence-based approach needed during the selection process, the use of clinical experience instead of scientific evidence, the strong influence of pharmaceutical representatives in medicine selection and the lack of pharmacoeconomic application, all of which could influence rational selection. Updating national essential medicines lists and standard treatment guidelines is critical to achieving UHC, as countries often use them as guides for priority-setting exercises associated with publicly funded health benefit packages. While many national essential medicines lists that specify where such medicines should be available focus on tertiary and quaternary care, shortages of specialists create barriers to receiving a diagnosis, prescription and renewal of prescriptions. As such, allocating Parkinson disease medicines at primary and secondary levels could address barriers to access.

Fourth, medicines that are not locally registered or authorized in a country do not have the licence for sale and distribution. Therefore, medicine registration and market authorization are key regulatory processes that enable access to medicines. However, regulatory processes for medicine registration can be complicated^39^ and many countries in the WHO African Region do not have sufficient capacity to provide extensive regulatory oversight, hence delaying access.^40^ The registration of medicines facilitates public procurement, which can enable access through public outlets at lower costs. Therefore, although the inclusion of medicines on national essential medicines lists is a key action to improve access, this step should be followed by registration.

The under-registration of essential medicines for Parkinson disease was determined in 2018.^41^ In Kenya and the United Republic of Tanzania, four of the five medicines for Parkinson disease on their national essential medicine lists were not registered; while in Uganda, the only medicine for Parkinson disease on the national essential medicines list was not registered. Furthermore, over-registration of non-essential medicines (which results in registering non-priority and sometimes clinically sub-optimal medicines) has been reported.^41^ Medicines are unlikely to generate interest to manufacturers if not included on national essential medicines lists and are, therefore, less likely to be registered.

Finally, the regional or local production of essential medicines is also worth considering. About 79% of pharmaceuticals consumed in Africa come from abroad,^42^ which can increase costs. The selection of essential medicines can be influenced by local availability, with the availability of medicines in the local market a criterion for inclusion in national essential medicines lists.^43^ However, barriers to regional or local production in the WHO African Region exist, including the high costs of raw materials,; lack of technical capacity to diversify their medicines manufacturing; lack of enabling policies; and challenges with infrastructure (e.g. interruptions to electricity supply).^44^

Despite these barriers, regional manufacturing should be considered. Given the high rate of underdiagnosis of Parkinson disease in the WHO African Region, current demand for medicines does not generate sufficient interest with local and international manufacturers, as costs may outweigh return on investment. Therefore, to increase demand, diagnostic and treatment rates for Parkinson disease should be improved through training health workers. Additionally, mechanisms for pooled procurement and global collaboration, and convergence and reliance mechanisms across regulatory agencies should be explored. These strategies could facilitate better access to medicines for Parkinson disease.

This study has limitations. First, we sourced national essential medicines lists mainly through internet searches or WHO contacts, as some government websites were not available or accessible to obtain the lists from there, and so there may be errors in some lists. Cross-referencing with WHO’s repository of national essential medicines lists ensured that we used the most up-to-date versions, although the repository may also have errors as it relies on sourcing lists by similar methods. Second, national essential medicines lists were in different formats and not standardized, which increased the risk of errors in data extraction. We minimized this risk by using a search function and having repeated analyses by different people. A next step would be to explore the registration status of medicines for Parkinson disease to understand whether the medicines listed on national essential medicines lists are also registered in the WHO African Region.

In conclusion, this analysis provides further evidence of the need for action to improve the accessibility of medicines for Parkinson disease in the African Region. In that regard, it highlights the important role that national essential medicines lists can play in procurement, prescribing and use of these medicines; lowering their costs; and raising awareness and political will for Parkinson disease.

Updating national essential medicines lists in the WHO African Region, through periodic revision following evidence-based methods and based on WHO’s essential medicines list and local epidemiological trends and priorities, offers an important step towards improving the accessibility of medicines for Parkinson disease. These medicines can significantly improve quality and length of life. This effort should be part of a comprehensive approach encompassing additional components, for example: registration with regulatory authorities; inclusion in UHC packages; increased awareness of Parkinson disease; and improved data on burden of disease, the social and financial burden of care for Parkinson disease and return on investment.
